# Assessing and Revising the Plan for Intelligence Testing

**DOI:** 10.3390/jintelligence2020029

**Published:** 2014-04-04

**Authors:** Evie Vergauwe, Nelson Cowan

**Affiliations:** 1 Department of Psychological Sciences, University of Missouri, McAlester Hall, Columbia, MO 65211 USA

**Keywords:** intelligence, working memory, individual differences

## Abstract

This brief commentary suggests that the usefulness of the concept intelligence might depend on how one defines intelligence and on whether one is using it for scientific versus practical purposes. Furthermore, it is suggested that the concept of working memory must not be overlooked when considering individual differences in intelligence.

Do we need the concept of intelligence? The answer to this question might depend on how one defines intelligence. One of the reasons to believe we do need the concept of intelligence is its use to differentiate between individuals and to predict future intelligent behavior of these individuals, with the criteria typically being long-term behaviors such as school and job performance. As mentioned in the editorial comment by de Boeck (this volume), the concept of intelligence seems inseparably linked to intelligence tests. Yet, these tests are just the part of performance that can be distilled into a brief session. As Hunt and Jaeggi (this volume) noted, performance also is likely to depend on longer-term qualities that we wish we could estimate easily. Specifically, they hoped we could measure “cognitive traits that can only be evaluated by observations over time, such as the ability to conduct reflexive thought or to consider a problem from multiple perspectives.” These, however, probably include not only information-processing qualities (e.g., attending to a task until it is completed; inhibiting distractions when necessary) but also emotional and social qualities (e.g., having enough confidence to continue working; staying motivated; getting help from others when it is needed). For practical purposes, it is not obvious that there is a need to cordon off information processing rather than trying harder to obtain brief proxies for all relevant traits. On a more theoretical note, expanding the definition of intelligence to include longer-term cognitive qualities inflates the concept of intelligence beyond what intelligence tests measure and raises the question of which cognitive traits are encompassed by the concept of intelligence and which cognitive traits, if any, are not.

Intelligence tests are now rather taken for granted, to the point that they are often used in scientific investigations as criteria rather than predictors of more practical criteria. Hunt and Jaeggi referred to the issue of defining those situations in which intelligence is considered causal to behavior as the criterion problem. Although intelligence tests can be scientifically useful in this way, we wonder whether different practical situations might require different definitions of intelligence. For example, Cattell [1] distinguished between crystallized intelligence (Gc) and fluid intelligence (Gf), the first referring to domain-specific knowledge and the latter to the ability to deal with new, cognitively demanding situations. Often, when focusing on intelligence as a criterion variable, knowledge seems to be excluded from definition and testing. In some ways, this matters little because Gc and Gf typically correlate highly and individuals with high Gf should have a greater ability to learn and acquire new knowledge. However, one can easily imagine that some jobs might require a great ability to handle new situations (Gf) while others might rely heavily on previously acquired, diverse knowledge (Gc), such as a salesman who needs to know enough to be able to establish rapport with many different kinds of people. The specific requirements of a job might even change over time. When using intelligence tests to predict intelligent behavior, does it make sense to use a single definition across criterion situations? It seems that the less one knows about the requirements of the job situation, the more it makes sense to measure intelligence as a predictor for success. Otherwise, more job-specific tests may make more sense.

When the study of intelligence is described as the study of “individual differences in cognitive power”, the concept of working memory must not be overlooked. Working memory is often described as the mental workspace that allows us to keep active some information over a brief period of time in the service of online cognition, and it has been shown that reasoning ability (considered to be equivalent to Gf) correlates highly with working memory capacity. In fact, it correlates so highly that some researchers wondered whether reasoning ability is (little more than) working memory capacity ([2], see also [3] for a paper on the strong connection between working memory and Gf). We do not argue that reasoning and working memory are the same but we do think that these concepts are important when trying to understand various kinds of human comprehension, reasoning, and problem-solving. For example, the complexity of an idea that one can understand might depend on how many elements must be conjoined to make up the idea [4]. A concept like the verb bring, for example, is a subtle one that is difficult for young children whose working memory has not yet matured. Bring must be differentiated from similar verbs such as give, take, and send by keeping in mind several discriminating factors at once.

Also, the editorial comment mentions that it is possible to “study development and change in the size and kind of differences, but that is almost never done”. While this might be true for intelligence, this kind of research certainly has been conducted within the working memory field, which has also included intelligence subtests [5,6]. Another object of this kind of study, in addition to working memory, has been processing speed [7]. Even when tasks are not timed explicitly, they often need to be completed within a certain time period. For example, it is far more valuable to think of a good point of discussion during a committee meeting rather than after it is over and a group decision has been reached. Time constraints have been shown to be important in working memory, presumably to rehearse or reactivate information quickly enough to prevent it from being lost from working memory (e.g., [8,9]).

Perhaps it is time to consider the traits of intelligence that can be beneficial for the human condition; there are ways to do a job that might make a difference between selfish self-interest and enlightened self-interest, the latter helping everyone. Among cognitive traits, we believe that enlightened thinking would be thinking that could overcome confirmation bias in order to get at the truth more quickly [10] and that would be wary of bias against recognizing evidence conflicting with one’s own views. Another such trait might be the ability to take an insight that has been learned in one domain and figure out how to apply it judiciously to a very different situation (Johnson, this volume). Such types of thinking may be partly trainable. In the case of “myside bias” there appears to be very little correlation with the results of conventional intelligence tests [11]. We believe that we should start to learn how to measure such traits and to include them in test batteries. Perhaps these traits will inevitably involve non-cognitive qualities, such as emotional control, conscientiousness, persistence, and motivation. We need to decide whether these qualities can be included or excluded in principle, and we need to know if they have enough stability over time, which is one clear strength of standard intelligence tests as Johnson (this volume) documented.

To conclude, the usefulness of the concept intelligence might depend on how one defines intelligence and this, in turn, might depend on whether one is using it for scientific versus practical purposes.
